# Serum Level of Homocysteine, Folate and Vitamin-B12 in Epileptic Patients Under Carbamazepine and Sodium Valproate Treatment: A Systematic Review and Meta-Analysis

**DOI:** 10.5812/ircmj.9690

**Published:** 2013-03-05

**Authors:** Fazel Gorjipour, Yasin Asadi, Nushin K. Osguei, Marjan Effatkhah, Ali Samadikuchaksaraei

**Affiliations:** 1 Physiology Research Center, Iran University of Medical Sciences, Hemmat Campus, Tehran, IR Iran; 2 Physiology Research Center, Semnan University of Medical Sciences, Semnan, IR Iran; 3 Eposcience Millennium Institute, Tehran, IR Iran; 4 Department of Neurology, Faculty of Medicine, Iran University of Medical Sciences, Tehran, IR Iran; 5 Department of Medical Biotechnology, Faculty of Allied Medicine, Iran University of Medical Sciences, Tehran, IR Iran; 6 Cellular and Molecular Research Center, Iran University of Medical Sciences, Tehran, IR Iran

**Keywords:** Epilepsy, Anticonvulsants, Atherosclerosis, Adverse Effects, Carbamazepine, Valproic Acid

## Abstract

**Background:**

Numerous studies have shown that long term treatment with anticonvulsants may be an important risk factor for the onset of atherosclerosis, or worsening of its symptoms. There are many contradictory reports regarding these effects.

**Objectives:**

We performed a systematic review and meta-analysis of the published studies in order to see whether the atherogenic outcomes could be related to any serum biochemical abnormalities.

**Materials and Methods:**

Published articles indexed in PubMed, ISI web of science, Science Direct and Scopus databases from 1990 to 2011 were retrieved using a comprehensive search strategy. After omitting the unrelated articles and duplicates, articles met the eligibility criteria for critical appraisal were included in the analysis. Data were summarized in standard data abstraction forms and subjected to analysis by STATA software.

**Results:**

Finally, ten published studies were included in the meta-analysis. Results showed that carbamazepine and sodium valproate consumption are associated with a significant elevation of the serum homocysteine levels. On the other hand, medication with carbamazepine is associated with a reduction of the level of folate in the serum and that of sodium valproate is associated with a reduction of serum level of vitamin B12.

**Conclusions:**

According to the results of this study, as carbamazepine and valproate sodium consumption can result in elevated serum levels of homocysteine and decreased levels of folate and vitamin B12, and the atherogenic effect of increased serum homocysteine level is well established, the patients under these medications should be monitored for possible atherogenic effects.

## 1. Background

Epilepsy is a major neurologic disorder which affects about five to ten percent of the people worldwide. It is a chronic and dynamic medical condition which requires long term, and usually lifelong, treatment with anticonvulsants (1). Epidemiological studies have shown that in adults suffering from epilepsy, risk of development of atherogenic ischemic heart disease (IHD) and fatal cardiovascular disease increase to 34% and 68%, respectively (2). The underlying etiology of atherosclerosis-related vascular disease in epileptic patients has not yet been fully addressed. The possible etiologic suggestions are factors like alterations in the homocysteine metabolisms, lipid profile aberrations, serum lipoprotein increment, and deficiency of thyroidal hormones (2, 3). Impairment of endothelial function is another possible mechanism, which has been presumed to be the result of epilepsy itself, or side effects of long term treatment with antiepileptic agents. This may induce the process of atherogenesis and result in arterial obstructive diseases leading to stroke, myocardial infarction, etc. (4). Among the serum biochemical changes, alterations of the serum homocysteine, folate, and B12 following anticonvulsant therapy and their relationship to atherosclerosis is a subject of controversy (5-8)as the metabolism of these biochemical factors are inter-related and deficiency of either folate or vitamin B12 increases the serum homocysteine level.

## 2. Objectives

In the current study, we attempted to perform a systematic review and meta-analysis to find out whether there is a relationship between anticonvulsant therapy and alterations of serum levels of homocysteine, folate and vitamin B12 as an underlying cause of increased risk of atherogenicity in patients receiving anticonvulsant therapy.

## 3. Materials and Methods

### 3.1. Search Strategy and Databases

In this systematic review and meta-analysis, we first retrieved all articles published from 1990 to 2011 in PubMed, ISI Web of Science, Scopus and Science Direct databases. Keywords included carbamazepine, sodium valproate, epilepsy, antiepileptic, anticonvulsant, folate, folic acid, vitamin B12, homocystein and atherosclerosis. For PubMed we also used the MeSH controlled vocabulary to improve the specificity of our search strategy. For other databases simple searches based on keywords in title and abstract was used. Language of search strategy was limited to English. After exclusion of duplicate results, publications from animal studies, and abstracts presented in scientific meetings, the articles were reviewed by two independent researchers, and those related to this study were chosen. Data was extracted from selected publications and recorded in standard data abstraction forms. Finally two independent researchers chose the articles which were to be included in the analysis according to the critical appraisal criteria. If there was any ambiguity in inclusion of a study, the opinion of a third reviewer would be sought. Observational studies on patients on antiepileptic medications, in which mean and standard deviation of serum homocysteine, folate and vitamin B12 were reported, and their full-texts were available in English were included in the analysis. Articles not in English or those not reporting the mean and standard deviation homocysteine, folate and vitamin B12 were excluded from study. If there was any ambiguity about the reported results, the authors were directly contacted for clarification. Data related to the type of study, date of study, age group of subjects, gender of subjects, country in which the research was conducted, number of subjects, drugs, outcome measures, and reported results were entered into a spreadsheet file and analyzed by STATA v.11 (College Station, TX, Stata Corporation).

### 3.2. Data analysis 

For analysis, standardized mean difference (SMD) of extracted data, and the mean command in the STATA software were used. Also, the Cochrane's Q test for assessment of studies heterogeneity and funnel plot and Egger’s regression method for evaluation of the publication bias were used. The significance level was set at P < 0.05. This study is a secondary research and does not include ethical codes for human samples. Accordingly, approval of the research ethics committee was not necessary for this study. However, the researchers were committed to follow all of the standards and regulations related to research ethics.

## 4. Results

**Figure 1.**
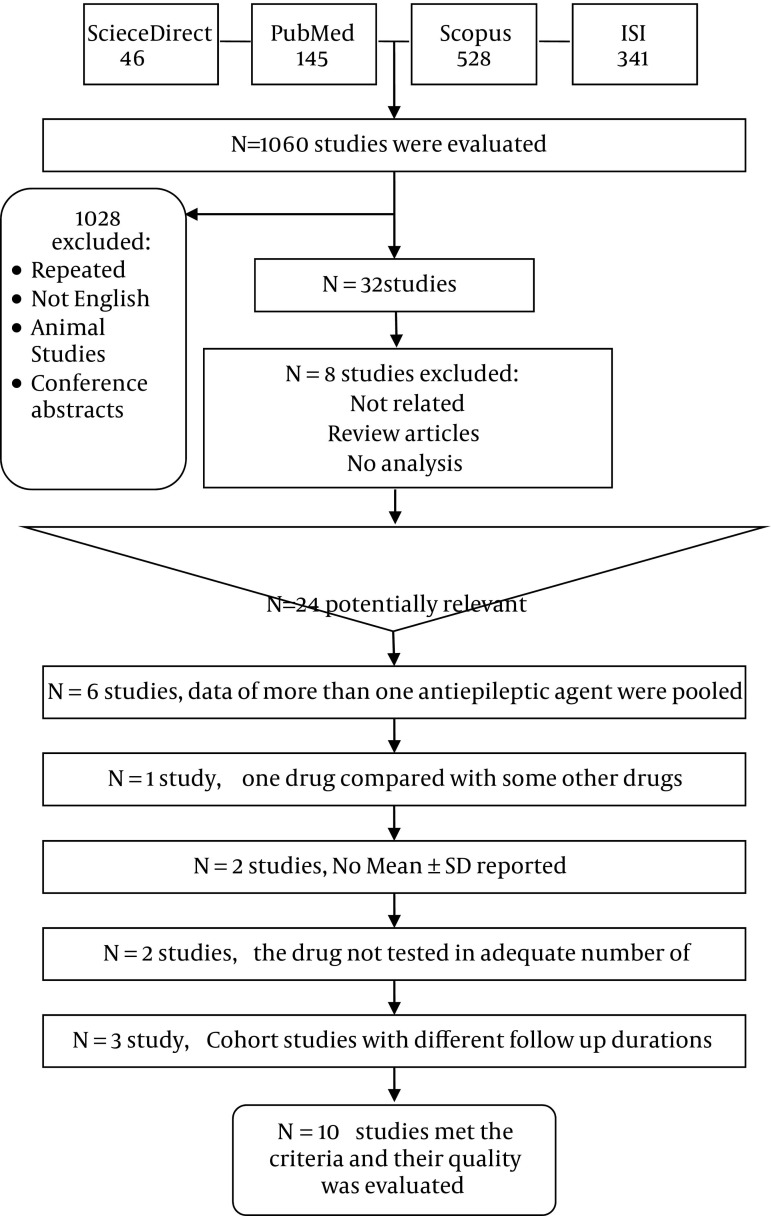
Flowchart depicts the process of selection of studies to be included in this analysis

**Figure 2:**
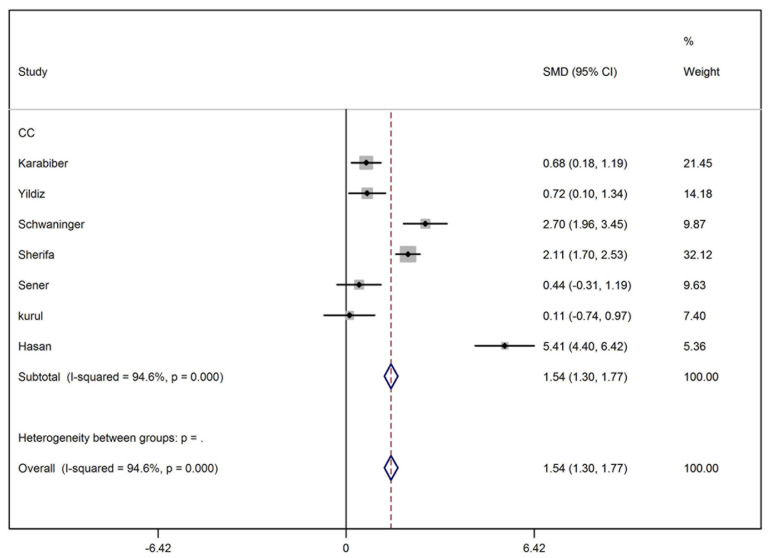
Forest Plot Representing the Effects of Carbamazepine on Blood Homocysteine Level

**Figure 3.**
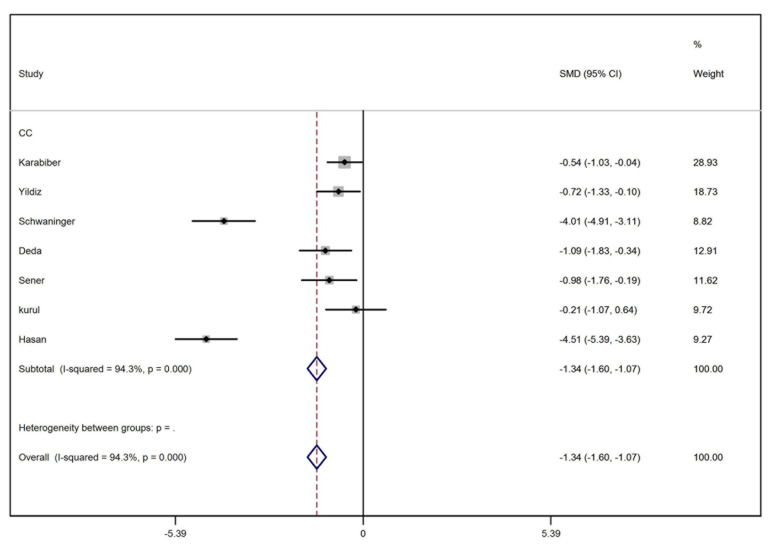
Forest Plot Representing the Effect of Carbamazepine on Blood-Folate Level

**Figure 4.**
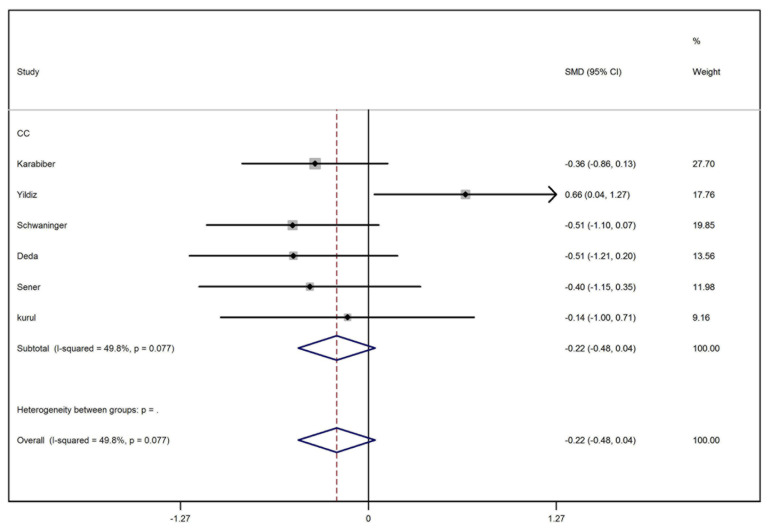
Forest Plot Representing the Effect of Carbamazepine on Blood Vitamin B12 Level

We retrieved 145 articles from PubMed, 341 articles from ISI Web of Knowledge, 528 articles from Scopus, and 46 articles from Science Direct. To sum up 1060 articles were retrieved and imported into EndNote software (v X.5, Thomson Reuters, New York, NY) where all duplicate results were discarded. Unrelated articles, conference abstracts and animal studies were deleted from the library. Thirty two articles were selected for a full-text assessment. After primary assessment of full-text articles, eight were omitted from the search results as they were unrelated to the topic or were review or case reports. Twenty four articles were thoroughly assessed again. In this step, 11 studies were excluded for the following reasons: the data of more than one antiepileptic agent was pooled, the mean or standard deviation was not reported and the study was comparing the effects of different antiepileptic (Figure 1). Three cohort studies were also excluded as their follow-up durations were different (20 weeks, 32 weeks and 12 months) and it was not possible to analyze their data. Finally, 10 studies were included in the analysis (Table 1). All included studies were of case-control type (Table 1). Results of meta-analysis and forest plots of the effect estimates for the relationship between receiving carbamazepine and the serum level of homocysteine, folate and vitamin B12 are represented in Figures 2, 3 and 4. Meta-analysis showed that the level of homocysteine was significantly higher in patients receiving carbamazepine (P < 0.001) (P for Heterogeneity ≤ 0.001; I-squared = 94.6%; no publication bias in funnel plot, no significant Egger’s test [P > 0.05]), while the serum folate levels were significantly lower than controls (P < 0.001) (P for Heterogeneity ≤ 0.001; I-squared = 94.3%; no publication bias in funnel plot, no significant Egger’s test (P > 0.05). Although, meta-analysis showed a reduction in the serum level of vitamin B12 in patients on carbamazepine, this reduction was not significant (P = 0.10) (I-squared = 49.8%; P for Heterogeneity = 0.08; no publication bias in funnel plot, no significant Egger’s test (P > 0.05) (Table 2). In regards to treatment with sodium valproate, the results showed that it was significantly associated with increased serum homocysteine levels (P < 0.001) (p for heterogeneity = 0.003; I-squared = 72.1%; no publication bias in funnel plot, no significant Egger’s test [P > 0.05]), but was not associated with reduction of the serum folate levels (P = 0.52) (p for heterogeneity = 0.01; I-squared=65.7%; no publication bias in funnel plot, no significant Egger’s test [P > 0.05]). On the other hand, a significant decrease in the serum levels of vitamin B12 was observed in patients receiving this medication (P ≤ 0.001) (p for heterogeneity = 0.2; I-squared = 31.9%; no publication bias in funnel plot, no significant Egger’s test (P > 0.05) (Table 2).

**Table 1.** List of studies included in the meta-analysis

References	Study	Country	Year	Study Type	Sample Size	Control	Gender	Age, y
**(9)**	Hamed	Egypt	2007	CC^a^	225	60	Male-Female	18-45
**(10)**	Schwaninger	Germany	1999	CC	51	51	Male-Female	49.1±13.9^a^
**(6)**	Karabiber	Turkey	2003	CC	66	29	Male-Female	2-16
**(3)**	Yildiz	Turkey	2010	CC	59	23	Male-Female	4-13
**(11)**	Deda	Turkey	2003	CC	16	16	Male-Female	5-19
**(12)**	Sener	Turkey	2006	CC	75	11	Male-Female	16-56
**(7)**	Kurul	Turkey	2007	CC	25	10	Male-Female	6-18
**(13)**	Hasan	Syria	2010	CC	45	28	Male-Female	6-64
**(14)**	Algin	Turkey	2009	CC	42	30	Male-Female	45.8±3.4^b^
**(15)**	Geda	Turkey	2002	CC	26	28	Male-Female	1-12

^a^Abbreviation: CC, case control

^b^This study presented the age as the Mean ± SD and did not mention the age range of the patients

**Table 2.** Results of the Meta-Analysis for Studies Reporting the Effects of Carbamazepine and Sodium Valproate on the Atherosclerosis Risk Factors

AED	Serum Level	Study, No.	SMD ^a^	95% Conf.	Interval	P value
**CBZ^a^**	Homocys^a^	7	1.54	1.30	1.77	< 0.001
	**Folate**	7	-1. 34	-1.60	-1.07	< 0.001
	**B12**	6	-0.22	-0.48	0.04	0.10
**Na Val^a^**	Homocys	6	0.76	0.51	1.02	< 0.001
	**Folate**	6	-0.09	-0.35	0.17	0.52
	**B12**	6	0.57	0.30	0.83	< 0.001

^a^Abbreviations: CBZ, Carbamazepine; Na Val, sodium valproate; SMD, standardized mean difference; Homocys, homocysteine

## 5. Discussion

Cardiovascular atherosclerotic diseases are amongst the most important causes of mortality worldwide. Although clinical manifestations do not occur until adulthood, pathologic changes start from childhood. Implication of the blood lipids aberrations in the pathogenesis of atherosclerosis is well-established (16, 17). Atherosclerosis is a multifactorial process. Possible prognostic factors include hyperlipidemia, high blood pressure, hyperglycemia, vitamins B6, B12 and folate deficiency and hyperhomocysteinaemia (18, 19). Homocysteine is a sulfur containing amino acid produced from metabolism of methionine, an essential amino acid. Elevated serum levels of this amino acid is a strong and independent predictor for increased risk of atherosclerosis development in coronary, aortic, carotid and peripheral arteries in a dose-dependent manner (20). The current study shows that receiving carbamazepine and sodium valproate is significantly associated with increased serum levels of homocysteine, which could be considered as the underlying cause of atherogenicity with consumption of these drugs. Since vitamins B6, B12 and folate are necessary for the metabolism of homocysteine to methionine; their deficiencies are amongst the most important causes of hyperhomocysteinemia. This might be the mechanism by which carbamazepine and sodium valproate increase the serum homocysteine level. On the other hand, it has been mentioned that some antiepileptics can lead to alterations of serum biochemicals such as cholesterol, lipoprotein and homocysteine through induction of cytochrome P450 (21). Changes in the serum level of thyroid hormones have also been mentioned as one of the possible mechanisms of atherogenicity of antiepileptic agents (2, 4, 22-24). However, the data collected from the articles evaluated in the current study did not consider serum lipids and thyroid hormone levels in a way suitable for inclusion in meta-analysis study. It should be noted that the majority of publications included in this study are from Turkey indicating that there is a high interest in this country to study variations of serum biochemical factors resulting from antiepileptic therapy. The low publication bias indices of this study confirm that no particular bias was involved in inclusion of publications from Turkey. However, more conclusive results could be drawn if measurements of the serum levels of homocysteine, folate and vitamin B12 following antiepileptic therapy could be conducted in populations with more diverse geographical and ethnical background. According to our results, the atherogenic effects of homocysteine and the association of hyperhomocysteinemia with the treatment of carbamazepine and sodium valproate indicate thatthese antiepileptics should be considered atherogenic. It is reasonable to carefully monitor patients receiving these drugs for potential atherogenic effects. Further prospective studies with adequate sample sizes are necessary to consolidate the results from this study.
